# 2-[(1-Methyl-1*H*-pyrrol-2-yl)methyl­idene]propane­dinitrile

**DOI:** 10.1107/S160053681201197X

**Published:** 2012-03-24

**Authors:** Abdullah M. Asiri, Hassan M. Faidallah, Shaeel A. Al-Thabaiti, Seik Weng Ng, Edward R. T. Tiekink

**Affiliations:** a Chemistry Department, Faculty of Science, King Abdulaziz University, PO Box 80203, Jeddah, Saudi Arabia; b The Center of Excellence for Advanced Materials Research, King Abdulaziz University, Jeddah, PO Box 80203, Saudi Arabia; c Department of Chemistry, University of Malaya, 50603 Kuala Lumpur, Malaysia

## Abstract

In the title compound, C_9_H_7_N_3_, the N-bound methyl group and vinyl H atom are *syn*. The 12 non-H atoms comprising the mol­ecule are essentially coplanar (r.m.s. deviation = 0.071 Å). Supra­molecular tapes feature in the crystal packing, orientated perpendicular to [10-1], and are formed by C—H⋯N inter­actions involving each cyano N atom. The tapes are connected into layers *via* π–π inter­actions occurring between translationally related pyrrole rings [ring centroid–centroid distance = 3.8754 (10) Å]; the layers stack along the *b* axis.

## Related literature

For the anti-cancer effects of related compounds, see: Rostom *et al.* (2011 ▶). For structural studies of di-carbonitrile compounds, see: Asiri *et al.* (2011 ▶); Al-Youbi *et al.* (2012 ▶).
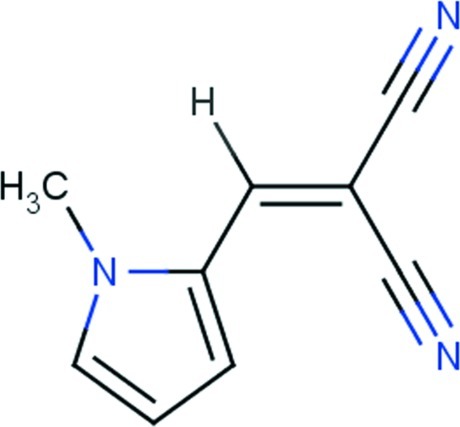


## Experimental

### 

#### Crystal data

C_9_H_7_N_3_
*M* *_r_* = 157.18
Triclinic, 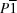

*a* = 3.8754 (2) Å
*b* = 8.7795 (5) Å
*c* = 12.1773 (7) Å
α = 97.517 (5)°
β = 90.962 (5)°
γ = 98.689 (5)°
*V* = 405.76 (4) Å^3^
*Z* = 2
Mo *K*α radiation
μ = 0.08 mm^−1^
*T* = 100 K
0.25 × 0.15 × 0.05 mm

#### Data collection

Agilent SuperNova Dual diffractometer with an Atlas detector
Absorption correction: multi-scan (*CrysAlis PRO*; Agilent, 2011 ▶) *T*_min_ = 0.980, *T*_max_ = 0.996
5871 measured reflections
1866 independent reflections
1463 reflections with *I* > 2σ(*I*)
*R*_int_ = 0.039

#### Refinement

*R*[*F* ^2^ > 2σ(*F* ^2^)] = 0.045
*wR*(*F* ^2^) = 0.124
*S* = 1.01
1866 reflections
137 parameters
All H-atom parameters refined
Δρ_max_ = 0.24 e Å^−3^
Δρ_min_ = −0.29 e Å^−3^

### 

Data collection: *CrysAlis PRO* (Agilent, 2011 ▶); cell refinement: *CrysAlis PRO*; data reduction: *CrysAlis PRO*; program(s) used to solve structure: *SHELXS97* (Sheldrick, 2008 ▶); program(s) used to refine structure: *SHELXL97* (Sheldrick, 2008 ▶); molecular graphics: *X-SEED* (Barbour, 2001 ▶) and *DIAMOND* (Brandenburg, 2006 ▶); software used to prepare material for publication: *publCIF* (Westrip, 2010 ▶).

## Supplementary Material

Crystal structure: contains datablock(s) global, I. DOI: 10.1107/S160053681201197X/bt5851sup1.cif

Structure factors: contains datablock(s) I. DOI: 10.1107/S160053681201197X/bt5851Isup2.hkl

Supplementary material file. DOI: 10.1107/S160053681201197X/bt5851Isup3.cml

Additional supplementary materials:  crystallographic information; 3D view; checkCIF report

## Figures and Tables

**Table 1:** Hydrogen-bond geometry (Å, °)

*D*—H⋯*A*	*D*—H	H⋯*A*	*D*⋯*A*	*D*—H⋯*A*
C3—H3⋯N3^i^	0.976 (19)	2.612 (19)	3.579 (2)	170.8 (16)
C6—H6⋯N2^ii^	0.969 (17)	2.515 (17)	3.469 (2)	167.8 (14)

Symmetry codes: (i) 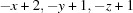; (ii) 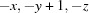.

**Fig. 1.**
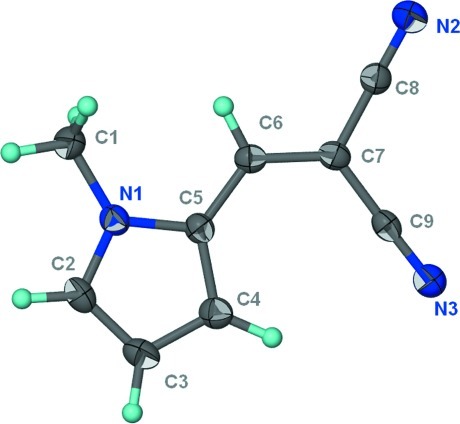
The molecular structure of (I) showing the atom-labelling scheme and displacement ellipsoids at the 70% probability level.

**Fig. 2.**
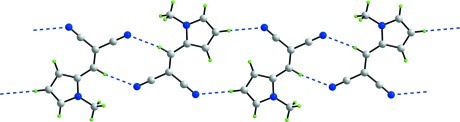
A view of the supramolecular tape in (I) with C—H···N interactions shown as blue dashed lines.

**Fig. 3.**
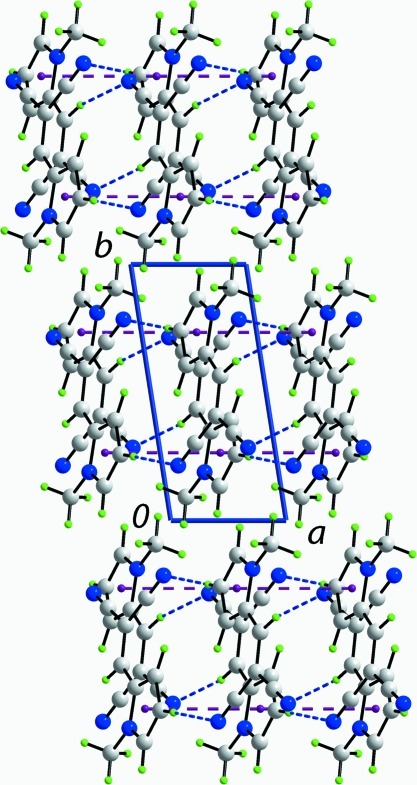
A view in projection down the c axis of the unit-cell contents of (I) showing the stacking of layers along the b axis. The C—H···N and π—π interactions are shown as blue and purple dashed lines, respectively.

## supplementary crystallographic information

### Comment

Arylidenes are considered as key intermediates for the synthesis of a variety of heterocycles of biological importance, such as pyridine, pyridazine and quinoline derivatives. Previous studies have shown that the derived compounds exhibit a variety of biological activities, including anti-cancer effects (Rostom *et al.*, 2011). In continuation of structural studies of di-carbonitrile compounds (Asiri *et al.*, 2011; Al-Youbi *et al.*, 2012), the title compound, (I), was investigated.

In (I), Fig. 1, the N-bound methyl group and vinyl-H atom are *syn*. The 12 non-hydrogen atoms are co-planar having a r.m.s. deviation = 0.071 Å, with the maximum deviations being 0.118 (2) Å for the C1 atom and -0.084 (2) Å for the N2 atom.

In the crystal packing, each cyano-N atom participates in a C—H···N interaction, Table 1, with a centrosymmetrically related molecule to form a supramolecular tape. The tape is orientated along [101] and comprises alternating 10-membered {···HC_3_N}_2_ and 16-membered {···HC_6_N}_2_ synthons, Fig. 2. The tapes are connected into layers *via*π—π interactions occurring between translationally related pyrrazole rings [ring centroid..centroid distance = 3.8754 (10) Å for symmetry operation 1 + *x*, *y*, *z*]. The layers stack along the *b* axis, Fig. 3.

### Experimental

A mixture of 1-methylpyrrole-2-carboxaldehyde (1.1 g, 0.01 mmol) and malononitrile (1.1 g, 0.01 mmol) in absolute ethanol (50 ml) was refluxed for 2 h. The reaction mixture was allowed to cool, and the formed precipitate was filtered, washed with water, dried and recrystallized from ethanol. Yield: 72%. *M*.pt: 427–229 K.

### Refinement

All H-atoms were located in a difference map and were refined freely, the range of C—H bond lengths = 0.952 (19) to 1.002 (19) Å.

### Crystal data

**Table:**

C_9_H_7_N_3_	*Z* = 2
*M**_r_* = 157.18	*F*(000) = 164
Triclinic, *P*1	*D*_x_ = 1.286 Mg m^−^^3^
Hall symbol: -P 1	Mo *K*α radiation, λ = 0.71073 Å
*a* = 3.8754 (2) Å	Cell parameters from 1724 reflections
*b* = 8.7795 (5) Å	θ = 2.4–27.5°
*c* = 12.1773 (7) Å	µ = 0.08 mm^−^^1^
α = 97.517 (5)°	*T* = 100 K
β = 90.962 (5)°	Prism, light-brown
γ = 98.689 (5)°	0.25 × 0.15 × 0.05 mm
*V* = 405.76 (4) Å^3^	

### Data collection

**Table:**

Agilent SuperNova Dual diffractometer with an Atlas detector	1866 independent reflections
Radiation source: SuperNova (Mo) X-ray Source	1463 reflections with *I* > 2σ(*I*)
Mirror monochromator	*R*_int_ = 0.039
Detector resolution: 10.4041 pixels mm^-1^	θ_max_ = 27.6°, θ_min_ = 2.4°
ω scan	*h* = −5→5
Absorption correction: multi-scan (*CrysAlis PRO*; Agilent, 2011)	*k* = −11→11
*T*_min_ = 0.980, *T*_max_ = 0.996	*l* = −15→15
5871 measured reflections	

### Refinement

**Table:**

Refinement on *F*^2^	Primary atom site location: structure-invariant direct methods
Least-squares matrix: full	Secondary atom site location: difference Fourier map
*R*[*F*^2^ > 2σ(*F*^2^)] = 0.045	Hydrogen site location: inferred from neighbouring sites
*wR*(*F*^2^) = 0.124	All H-atom parameters refined
*S* = 1.01	*w* = 1/[σ^2^(*F*_o_^2^) + (0.0531*P*)^2^ + 0.1622*P*] where *P* = (*F*_o_^2^ + 2*F*_c_^2^)/3
1866 reflections	(Δ/σ)_max_ = 0.001
137 parameters	Δρ_max_ = 0.24 e Å^−^^3^
0 restraints	Δρ_min_ = −0.29 e Å^−^^3^

### Fractional atomic coordinates and isotropic or equivalent isotropic displacement parameters (Å^2^)

**Table:**

	*x*	*y*	*z*	*U*_iso_*/*U*_eq_	
N1	0.3583 (3)	0.18886 (15)	0.26433 (10)	0.0202 (3)	
N2	0.2317 (4)	0.71488 (16)	0.02337 (11)	0.0267 (3)	
N3	0.8617 (4)	0.78536 (16)	0.32981 (11)	0.0267 (3)	
C1	0.1644 (5)	0.0922 (2)	0.16953 (14)	0.0250 (4)	
C2	0.4697 (4)	0.13538 (19)	0.35574 (13)	0.0234 (4)	
C3	0.6539 (4)	0.25848 (19)	0.42647 (13)	0.0246 (4)	
C4	0.6548 (4)	0.39208 (19)	0.37650 (12)	0.0220 (4)	
C5	0.4681 (4)	0.34899 (17)	0.27400 (12)	0.0188 (3)	
C6	0.3763 (4)	0.43469 (17)	0.19062 (12)	0.0185 (3)	
C7	0.4655 (4)	0.59152 (18)	0.18566 (12)	0.0190 (3)	
C8	0.3370 (4)	0.65898 (17)	0.09541 (12)	0.0202 (3)	
C9	0.6828 (4)	0.69787 (17)	0.26673 (12)	0.0196 (3)	
H11	0.106 (5)	−0.015 (3)	0.1842 (17)	0.041 (6)*	
H12	0.308 (5)	0.090 (2)	0.1053 (17)	0.038 (5)*	
H13	−0.050 (5)	0.132 (2)	0.1524 (16)	0.033 (5)*	
H2	0.412 (5)	0.027 (2)	0.3608 (15)	0.025 (4)*	
H3	0.763 (5)	0.250 (2)	0.4978 (16)	0.031 (5)*	
H4	0.769 (5)	0.500 (2)	0.4051 (14)	0.024 (4)*	
H6	0.220 (4)	0.378 (2)	0.1311 (14)	0.020 (4)*	

### Atomic displacement parameters (Å^2^)

**Table:**

	*U*^11^	*U*^22^	*U*^33^	*U*^12^	*U*^13^	*U*^23^
N1	0.0225 (7)	0.0198 (7)	0.0189 (6)	0.0031 (5)	0.0018 (5)	0.0047 (5)
N2	0.0320 (8)	0.0269 (7)	0.0215 (7)	0.0040 (6)	−0.0007 (6)	0.0056 (6)
N3	0.0310 (8)	0.0241 (7)	0.0245 (7)	0.0008 (6)	−0.0021 (6)	0.0062 (6)
C1	0.0282 (9)	0.0221 (8)	0.0235 (8)	0.0002 (7)	−0.0013 (7)	0.0037 (6)
C2	0.0255 (8)	0.0227 (8)	0.0244 (8)	0.0054 (6)	0.0050 (6)	0.0097 (6)
C3	0.0254 (8)	0.0301 (9)	0.0201 (8)	0.0054 (7)	0.0008 (6)	0.0089 (6)
C4	0.0209 (8)	0.0249 (8)	0.0204 (8)	0.0024 (6)	0.0018 (6)	0.0046 (6)
C5	0.0188 (7)	0.0196 (7)	0.0182 (7)	0.0022 (6)	0.0029 (6)	0.0045 (6)
C6	0.0179 (7)	0.0213 (8)	0.0163 (7)	0.0034 (6)	0.0018 (6)	0.0023 (6)
C7	0.0195 (7)	0.0221 (8)	0.0162 (7)	0.0042 (6)	0.0023 (6)	0.0041 (6)
C8	0.0214 (8)	0.0201 (7)	0.0191 (8)	0.0028 (6)	0.0022 (6)	0.0023 (6)
C9	0.0208 (7)	0.0201 (7)	0.0198 (8)	0.0041 (6)	0.0037 (6)	0.0079 (6)

### Geometric parameters (Å, º)

**Table:**

N1—C2	1.352 (2)	C3—C4	1.390 (2)
N1—C5	1.3949 (19)	C3—H3	0.977 (19)
N1—C1	1.463 (2)	C4—C5	1.410 (2)
N2—C8	1.157 (2)	C4—H4	1.002 (19)
N3—C9	1.152 (2)	C5—C6	1.412 (2)
C1—H11	0.97 (2)	C6—C7	1.378 (2)
C1—H12	0.97 (2)	C6—H6	0.969 (17)
C1—H13	0.98 (2)	C7—C8	1.431 (2)
C2—C3	1.386 (2)	C7—C9	1.431 (2)
C2—H2	0.952 (19)		
			
C2—N1—C5	108.97 (13)	C3—C4—C5	107.69 (14)
C2—N1—C1	124.98 (13)	C3—C4—H4	127.8 (10)
C5—N1—C1	126.02 (13)	C5—C4—H4	124.5 (10)
N1—C1—H11	110.6 (12)	N1—C5—C4	106.64 (13)
N1—C1—H12	109.7 (11)	N1—C5—C6	120.42 (13)
H11—C1—H12	106.9 (17)	C4—C5—C6	132.91 (14)
N1—C1—H13	111.0 (11)	C7—C6—C5	128.23 (14)
H11—C1—H13	109.4 (17)	C7—C6—H6	115.2 (10)
H12—C1—H13	109.2 (16)	C5—C6—H6	116.5 (10)
N1—C2—C3	109.17 (14)	C6—C7—C8	120.18 (13)
N1—C2—H2	118.3 (11)	C6—C7—C9	124.54 (13)
C3—C2—H2	132.5 (11)	C8—C7—C9	115.29 (13)
C2—C3—C4	107.53 (14)	N2—C8—C7	179.15 (16)
C2—C3—H3	125.1 (11)	N3—C9—C7	178.23 (16)
C4—C3—H3	127.3 (11)		
			
C5—N1—C2—C3	−0.11 (17)	C1—N1—C5—C6	3.9 (2)
C1—N1—C2—C3	177.99 (14)	C3—C4—C5—N1	−0.10 (17)
N1—C2—C3—C4	0.05 (18)	C3—C4—C5—C6	177.75 (16)
C2—C3—C4—C5	0.04 (18)	N1—C5—C6—C7	−178.89 (14)
C2—N1—C5—C4	0.13 (17)	C4—C5—C6—C7	3.5 (3)
C1—N1—C5—C4	−177.94 (14)	C5—C6—C7—C8	−177.93 (14)
C2—N1—C5—C6	−178.05 (13)	C5—C6—C7—C9	1.6 (2)

### Hydrogen-bond geometry (Å, º)

**Table:**

*D*—H···*A*	*D*—H	H···*A*	*D*···*A*	*D*—H···*A*
C3—H3···N3^i^	0.976 (19)	2.612 (19)	3.579 (2)	170.8 (16)
C6—H6···N2^ii^	0.969 (17)	2.515 (17)	3.469 (2)	167.8 (14)

Symmetry codes: (i) −*x*+2, −*y*+1, −*z*+1; (ii) −*x*, −*y*+1, −*z*.
